# The Multifaceted Aspects of Interstitial Lung Disease in Rheumatoid Arthritis

**DOI:** 10.1155/2013/759760

**Published:** 2013-09-25

**Authors:** Lorenzo Cavagna, Sara Monti, Vittorio Grosso, Nicola Boffini, Eva Scorletti, Gloria Crepaldi, Roberto Caporali

**Affiliations:** Division of Rheumatology, University and IRCCS Foundation Policlinico S. Matteo, Viale Golgi 3, 27100 Pavia, Italy

## Abstract

Interstitial lung disease (ILD) is a relevant extra-articular manifestation of rheumatoid arthritis (RA) that may occur either in early stages or as a complication of long-standing disease. RA related ILD (RA-ILD) significantly influences the *quoad vitam* prognosis of these patients. Several histopathological patterns of RA-ILD have been described: usual interstitial pneumonia (UIP) is the most frequent one, followed by nonspecific interstitial pneumonia (NSIP); other patterns are less commonly observed. Several factors have been associated with an increased risk of developing RA-ILD. The genetic background plays a fundamental but not sufficient role; smoking is an independent predictor of ILD, and a correlation with the presence of rheumatoid factor and anti-cyclic citrullinated peptide antibodies has also been reported. Moreover, both *exnovo* occurrence and progression of ILD have been related to drug therapies that are commonly prescribed in RA, such as methotrexate, leflunomide, anti-TNF alpha agents, and rituximab. A greater understanding of the disease process is necessary in order to improve the therapeutic approach to ILD and RA itself and to reduce the burden of this severe extra-articular manifestation.

## 1. Introduction

Rheumatoid arthritis (RA) is a chronic, inflammatory condition that mainly affects joints, in terms of pain, erosion, disability, and reduced survival [1–5]. RA may be complicated by several extra-articular manifestations (EAMs) [6–8]. The lung is among the most important targets of EAMs; the spectrum of lung involvement in RA includes manifestations such as pleural disease, rheumatoid nodules, Caplan's syndrome, bronchiectasis [9, 10] and, in particular, interstitial lung disease (ILD) [11–14]. The first report of a correlation between pulmonary fibrosis and RA was published in 1948 by Ellman and Ball [11], describing three patients with polyarthritis and interstitial pneumonitis with chronic fibrosing aspects on autopsy. Since this first description as a “curious chronic fibrosing bronchopneumonic lesion,” it has become clear that RA associated interstitial lung disease (RA-ILD) actually includes a broad spectrum of disorders that vary greatly in their clinical presentation, pathology, and prognosis [9]. In the following years several authors pointed out the relevance of RA-ILD [15, 16], now widely accepted as an extra-articular complication with deep impact on prognosis and on therapeutic approach to RA [17–19]. Several histopathological patterns of ILD have been described [17] and differential diagnosis may be troublesome [20]. The etiopathogenesis of RA-ILD is not completely understood although genetic [21, 22], humoral [23], and environmental [24] factors seem to be involved. The picture is further complicated by the possible ILD-promoting effect of several drugs used to treat RA such as DMARDs (e.g., methotrexate and leflunomide) [25, 26] and biological agents (e.g., anti-TNF alpha and rituximab) [27, 28]. In this review we evaluated the main clinical characteristics of RA-ILD, the possible mechanisms underlying the occurrence of this EAM, and the current therapeutic approach.

## 2. Epidemiology and Prognosis

There is a great variation in the estimates of occurrence and in the general aspects associated with RA-ILD; this is partly due to the lack of acknowledged terminology and validated classification criteria, and to the different means of detection employed to diagnose ILD. In 2002, the American Thoracic Society and European Respiratory Society (ATS/ERS) redefined the nomenclature now used for acute and chronic diffuse parenchymal lung diseases [29]. Because of the lack of a dedicated classification, the consensus classification for idiopathic interstitial pneumonias (IIPs) has been adopted to define RA-ILD [30, 31]. RA-ILD can present as any of the seven idiopathic interstitial pneumonias according to the ATS/ERS consensus classification. Usual interstitial pneumonia (UIP) and nonspecific interstitial pneumonia (NSIP) are the main patterns of ILD described in RA although also other forms, including lymphocytic interstitial pneumonia (LIP) and organizing pneumonia (OP), have been less commonly observed [14, 31, 32]. In Table 1 we summarized the different patterns of ILD that may be detected in RA.

The predominance of UIP distinguishes RA from the majority of other connective tissue diseases (CTDs), usually characterized by a prevalence of NSIP pattern [20, 33] although a bias related to diagnostic methodologies is possible. Diagnostic accuracy of high resolution computed tomography (HRCT) for UIP and NSIP with respect to histological diagnosis has been reported to be approximately 70% in various studies, with possible discordance in up to one-third of cases [34]. Despite these limits, the estimates of RA-ILD prevalence likely fall in the range from 4% to 30% [6, 14, 35], depending on detection methods and selection criteria, and the incidence may be as high as 4.1 per 1,000 people with RA [17].

Although RA is predominant in females, RA-ILD is frequently described in males [9, 10, 19, 31], with a 2 : 1 male to female ratio [10]. It is interesting to observe that even if the incidence of severe EAMs, such as vasculitis, has decreased in the last decades, fewer changes have been observed for ILD [36, 37]. ILD is frequently an early and asymptomatic finding in RA; in a recent study up to 27% of patients had HRCT findings of ILD within 2 years from disease onset [38], whereas in another study 25% of RA patients had ILD already diagnosed at presentation and another 25% developed ILD within 3 years from disease onset [17]. 

RA-ILD is a significant cause of mortality, with a median survival of 2.6 years versus 9.9 years of RA patients without ILD. The standardized mortality ratio for RA-ILD compared to RA alone is 2.86 [19]. The increased mortality in RA-ILD is mainly due to ILD progression with respiratory failure and direct RA complications [39]. Infectious processes, lung cancer, pulmonary embolisms, and other non-ILD pulmonary conditions, although reported in the setting of RA-ILD [40], are not so relevant on mortality rates as theoretically conceivable [39]. The literature data show that ILD contributes approximately to 6–13% of the excess mortality of RA patients when compared to the general population [19, 39, 41], being one of the most significant causes of death in these patients, together with cardiovascular complications [7, 42]. 

Despite these data, ILD associated with collagen vascular diseases, including RA, was reported to have a better prognosis than the idiopathic type of ILD [9, 31], even in cases of RA patients with biopsy proven UIP [42]. A median survival of 60 months in RA-ILD compared with 27 months in IPF has been reported [13]. However, this latter point is a matter of debate; according to different authors the prognosis of RA-UIP patients does not differ from that of IPF [11]. Possible factors responsible for these contradictory results could be the occurrence of subtle histologic differences not detectable by conventional radiographic techniques but influencing the correct diagnosis [34], the positive or detrimental influence of immunosuppressive therapies used in the treatment of RA, and the systemic consequences of a chronic autoimmune disease such as RA. Differences in prognosis among RA-ILD patients with different patterns of pulmonary involvement have also been described. Several studies have suggested a relationship of the UIP pattern to a shorter survival particularly when compared to NSIP [10].

## 3. Risk Factors 

Several environmental, serologic, clinical, and genetic factors have been associated with the occurrence of RA-ILD. Occupational exposure to inhaled pollutants such as silica may lead to chronic lung inflammation and to the development of various autoimmune diseases such as RA [43]. In established RA, however, silica exposure is associated with the so called “Caplan's syndrome,” first described in the early fifties [44]; this is a peculiar syndrome characterized by the occurrence of multiple well-defined rounded nodules on chest X-ray, of various diameters, distributed throughout the lungs but predominantly at the lung periphery [44, 45]. Lesions appear often in crops may coalesce and form a larger confluent nodule that often may cavitate or calcify [44, 45]. In subjects not affected by RA, silica exposure is associated with a large amount of pathological lung conditions, such as pneumoconiosis, a peculiar form of interstitial lung disease that may lead to progressive massive fibrosis; exposure to silica and coal mine dusts may also result in pulmonary scarring in a pattern that mimics idiopathic pulmonary fibrosis [46]. To date, the pathogenetic link between exposure to silica, pneumoconiosis, Caplan's syndrome, and RA has not been clarified conclusively.

Smoking represents an independent risk factor for the occurrence of both autoantibody-positive [47, 48] and -negative RA [48]. The correlation is particularly evident for heavy (≥10 pack-years) and currently smoking individuals [48, 49]. Smoking is associated with several lung morbidities, including ILD [50–52]. The association with smoking is particularly evident for the UIP pattern [30, 53–55]. However, in a recent study involving 356 RA patients with lung involvement (either ILD or airway disease), the strong association between smoking history and ILD observed in the univariate analysis was not confirmed in the multinomial logistic regression analysis, thus suggesting that factors other than smoking may trigger ILD occurrence in RA [23]. According to this study, possible risk factors for RA-ILD are high titers of rheumatoid factor (RF), and to a lesser extent, anticyclic citrullinated peptide antibodies (ACPA), carriage of HLA-DRB1*1502, and older age [23]. The association of an increased risk of RA-ILD in RF positive patients has been confirmed by other studies [38, 56]. Data regarding the effect of ACPA on the development of lung disease are contradictory, with some authors confirming the association [57, 58] and others denying a possible role in the development of RA-ILD [59]. It is interesting to observe that both RF and ACPA can be present in smokers with ILD without clinical evidence of RA [60, 61]. Furthermore citrullination processes have been observed in lung tissue obtained from RA-ILD [62], and peculiar isoforms of citrullinated peptides, as, for example, the Hsp90 Isoforms [63], may be important antigen targets in RA-ILD. The increased citrullination of proteins and peptides in the lung is potentially smoke related due to peptidylarginine deiminase (PAD) increased activity and seems to play a role not only in ILD but also in RA occurrence [64]. The association with an allele connected with RA-ILD development, but not with occurrence of RA itself, HLA-DRB1*1502 has been reported in a different study [65]. As recently described, RA patients carrying DR2 serology (HLADRB1*15 and 16 alleles) and DQB1*06 have an increased risk of ILD, whereas HLA-DRB1 Shared Epitope (SE) appears to be protective against ILD development [66]. The protective role of HLA-DRB1 SE is indeed an unexpected finding, being generally associated with an increased risk of severe EAMs in RA [67, 68]. It should be underlined that these results have been observed in Japanese patients in whom the HLA allelic distribution is quite different from that of other ethnicities; therefore, these associations [23, 65, 66] should be confirmed by further studies. Older age has also been reported as a possible risk factor for the development of ILD [17, 19, 23]. Koduri et al. reported that risk estimates calculated on a 10-year difference in age are associated with a 64% increase in the likelihood of a RA patient of developing ILD over a shorter period of time [17]. Similar results were obtained by Bongartz et al. [19] and by Mori et al. [23]. Although the reason for this association is not completely clear, it is probably related to the established evidence of an increased incidence of EAMs in older RA patients (≥60 years) compared to younger ones [36, 69, 70].

Other risk factors associated with RA-ILD have been reported: high RA disease activity (e.g., DAS28 score) [19, 38], high grade functional impairment (measured using health assessment questionnaire) [17], and the presence of articular erosions and rheumatoid nodules [19].

## 4. DMARDs and Biological Agents Related ILD

In clinical practice, one of the most controversial issues is the possibility of ILD-promoting effects of some RA therapeutics. In fact, drugs such as methotrexate (MTX), leflunomide (LEF), antitumor necrosis factors (TNF) alpha agents, and rituximab have been associated with ILD occurrence or progression. 

MTX may cause ILD/pneumonitis in 0.86%–6.9% of patients with morbidity and mortality rates reaching 20% [71]. As recently reported, this side effect may be triggered by genetic factors; HLA-A*31:01 allele has been reported to be a possible predictor of MTX induced ILD in Japanese patients [72]. This particular genetic subset is more common in Japanese population compared to the Caucasian one (8.7% versus 3.9%), possibly explaining the increased risk of this drug side effect in Japan [73]. Even if environmental factors may be involved [74], hypersensitivity is probably responsible for most cases of pneumonitis associated with MTX, particularly in the case established lung disease [55, 75]. 

 Leflunomide is also associated with ILD [76], with an increased risk in the setting of preexisting lung disease and a potential relevant impact on survival [77, 78]. Similarly to MTX, hypersensitivity reactions may represent the main mechanism of LEF-induced pneumonitis [79]. The development of interstitial fibrosis is possibly connected with the effect of A771726, an active metabolite of LEF that may induce the transition of lung epithelial cells to myofibrasts [80], a phenomenon known as *“epithelial-mesenchymal transition” *(EMT). EMT is usually involved not only in abnormal wound repair and tissue remodelling, but also in organ fibrosis and reasonably in ILD [81]. However, EMT is not the only pathogenetic mechanisms in LEF-induced ILD. Experimental animal models demonstrated that the administration of LEF alone did not induce the EMT phenotype, but when LEF was administrated in the setting of a profibroting environment, such as bleomycin-induced pulmonary fibrosis, EMT was enhanced. This evidence suggests that the presence of other fibrosis-inducing stimuli, like in the case of preexisting lung fibrosis, may act as important risk factors for the development of LEF-induced ILD [80]. 

In recent years an increased number of reports described the new-onset or exacerbation of ILD after administration of biologic therapies, in particular anti-TNF alpha agents [82–84]. These drugs are pivotal in the treatment of several conditions, in particular RA [85, 86]. ILD may occur throughout the entire course of therapy with these agents, with reports ranging from few months to several years after treatment is introduced, with a mean interval of approximately 26 weeks. A possible correlation with RA-ILD has been reported for all anti-TNF alpha agents approved for the treatment of RA (infliximab, etanercept, adalimumab, certolizumab, and golimumab) [83, 87, 88]. The mechanism of anti-TNF-alpha induced ILD is not well established. TNF-alpha is usually considered as a key cytokine in the pathogenesis of interstitial pneumonia [89–91]. Paradoxically, anti-TNF agents may actually have profibrotic effects on the lung. In fact, TNF-alpha promotes apoptosis of pulmonary inflammatory cells, thereby mediating tissue healing. Anti-TNF-alpha therapies may inhibit these apoptotic processes and promote persistence of inflammatory cells in the lung parenchyma and eventually the development of ILD [92]. Support for this hypothesis originates from experiments on TNF-alpha homozygous knockout mice in which intratracheal administration of bleomycin resulted in an accelerated form of pulmonary fibrosis. Exposing these mice to recombinant TNF-alpha effectively reduced inflammation and promoted lung tissue healing [93]. Anti-TNF therapy also promotes the expression of antiinflammatory cytokines, such as TGF-*β*1, potentially contributing to profibrotic states [94]. Finally, considering the role of TNF-alpha gene polymorphisms in determing the effectiveness of anti-TNF alpha therapy [95, 96], it is tempting to speculate that a similar mechanism may be involved in the susceptibility to drug-induced ILD, but further studies are needed to confirm this hypothesis.

Anti-TNF induced ILD may present with different patterns of interstitial involvement, most commonly UIP or NSIP; cases of organizing pneumonia, diffuse alveolar damage, and lymphoid interstitial pneumonia have also been described. Complete resolution may be observed in up to 40% after withdrawal of the biologic agent [83]. Mortality can be as high as 30% of cases, rising to about 60% in case of preexisting ILD [83]. Older age (>60–65 years) has been reported as a negative prognostic factor [83, 88]. According to data derived from one European biologics registry, general mortality in RA-ILD does not seem to be different between patients treated with conventional DMARDs or with anti-TNF-alpha agents although the proportion of deaths directly attributable to ILD is reported to be higher in this latter group [97]. Further studies on higher numbers of patients are required to confirm these results. 

Despite the previously mentioned evidence, the association of anti-TNF alpha treatment and ILD development has been recently questioned. A large study including 8417 patients affected by RA, ankylosing spondylitis, psoriatic arthritis, psoriasis, and inflammatory bowel disease did not show an increased rate of ILD among the 4200 patients treated with anti-TNF agents, compared to those treated with non-biologic therapies, particularly in the RA group (adjusted hazard ratio, 1.03; 95% CI 0.51–2.07) [98]. Reports also suggest a possible improvement of RA-ILD following treatment with infliximab [99–101] and etanercept [102, 103]. The peculiar and nonunivocal link between anti-TNF alpha agents and ILD is clearly represented by a patient described by Komiya et al.; in this case report the administration of adalimumab first improved preexisting RA-ILD, and then induced its progression [104]. 

Finally, also other biological therapies with different mechanisms of action, including rituximab, have been involved in the occurrence of ILD in RA patients [10]. Some authors described the occurrence *exnovo* of ILD, following rituximab infusion [28]. ILD has also been reported during rituximab therapy for lymphoproliferative disorders [105]. The pathogenetic mechanism is not established. It is possible that by targeting CD20 positive cells, rituximab induces B-cell apoptosis, leading to antigen-presenting-cell maturation, cytotoxic T-cell activation, and subsequent vascular and alveolar damage [106]. In rituximab-ILD cytokine profile shows an increase of proinflammatory molecules such as IL-6 and TNF-alpha, with potential pathogenetic role in promoting interstitial fibrosis [107].

## 5. RA-ILD: Potential Pathogenic Cascade

The pathogenesis of RA-ILD is far from being completely clarified. Several multifactorial components and a large number of risk factors may be involved. Patients' genetic asset could be either predisposing (HLADRB1*15, HLADRB1*16, DQB1*06 [23, 66], and HLA-A*31:01 [72] alleles, this latter for MTX-related ILD) or protective (HLA-DRB1 SE [66]) for the development of RA-ILD. Environmental factors play a crucial role on a susceptible genetic background. Tobacco use has been recognized as a possible trigger in the development of RA-ILD. Smoking may directly injure respiratory epithelia and vascular endothelial cells [108, 109] and stimulate protein citrullination in the lung through local activation of PAD enzymes [64]. Citrullinated proteins may be key elements already at a preclinical level, acting as antigen targets for the local immune response, eventually leading to ACPA formation, RA occurrence [110], and finally lung interstitial involvement, characterized by particularly enhanced citrullination processes [23, 62, 63]. Furthermore, as previously mentioned, smoking may induce RF synthesis [111, 112] that has been associated with an increased risk of RA-ILD by several authors [19, 37, 41]. It is conceivable that smoking may trigger pulmonary immune response through direct lung damage, enhancement of protein citrullination, and autoantibodies synthesis (e.g., RF and ACPA). These events stimulate a local inflammatory response characterized by cellular infiltration and by the release of several mediators that further contribute to tissue damage [14, 113]. It must be considered that smoking is obviously not the only etiologic agent involved in the development of the disease as demonstrated by the fact that RA and RA-ILD often occur in nonsmokers. This suggests that other environmental [114] or infectious [14] factors are probably involved. Moreover, as analyzed in a previous section, drug-induced ILD is another relevant issue. 

Pulmonary cellular infiltrate in RA-ILD is heterogeneous, frequently organized in lymphoid nodular aggregates [115], showing striking similarities with rheumatoid synovium [116]. Follicular B lymphocyte aggregates are mainly peribronchiolar in disposition [117], resembling bronchial-associated lymphoid tissue. These structures are involved in antigen retrieval directly from lung lumen and not through afferent lymphatics as in lymph nodes and are capable of mounting adaptive immune responses [117]. Interestingly, an increased number of peribronchiolar B-cell follicles have been correlated with smoking [117], supporting once again not only the possible role of the lung and of environmental factors in promoting RA-ILD, but also RA itself [64, 117]. Follicular structures are generally accompanied by diffuse infiltration of pulmonary interstitium by plasma cells that may contribute to the humoral immune response through the production of ACPA [117]. These antibodies complex with citrullinated peptides through Fc receptors expressed on antigen presenting cells, ultimately leading to the production of proinflammatory cytokines, including TNF-alpha [64]. T-lymphocytes are essential to support B-cells activation and differentiation following antigen exposure. An increased CD4+ and, to a lesser extent, CD3+ T cells infiltrate has been demonstrated in RA-ILD, particularly compared to IIP, independently of the pattern of disease presentation (e.g., RA-UIP or RA-NSIP) [118]. Other authors suggested that CD8+ T cells may also be important in the development of pulmonary fibrosis in RA [119]. Although this hypothesis is supported by a report showing that smoking increases the number of CD8+ T cells in lungs [120], this plausible mechanism of injury should still be elucidated [119]. Furthermore, also neutrophils may be relevant in RA-ILD as suggested by the correlation of bronchoalveolar lavage neutrophilia and evidence of more advanced interstitial fibrosis [119, 121]. The complexity of lung cellular infiltrates has been confirmed in a recent model of RA-ILD in SKG mice: infiltrating cells observed in this model included CD4+ T cells, B cells, macrophages, and neutrophils [122]. Cytokines and chemokines play a crucial role in the interaction and crosstalk of the cellular network in RA-ILD. TNF-alpha is a key proinflammatory cytokine in the pathogenesis of interstitial lung involvement; it is mainly produced by activated macrophages, lymphocytes, epithelial, and endothelial cells. TNF-alpha plays a central role in the stimulation of cell-cell adhesion and transendothelial migration with a pivotal role in the early phases and in the maintenance of cytokines and chemokines production cascade [123]. TNF-alpha stimulates fibroblasts proliferation promoting their ability to degrade the extracellular matrix [124] and triggering the expression of growth factors such as platelet derived growth factor-*β* (PDGF-*β*) and transforming growth factor-*β* (TGF-*β*), cytokines such as interleukin-4 (IL-4) and interleukin-13 (IL-13), and chemokines (e.g., CXCL5, CXCL8, CXCL12, and CXCL13) [14, 64, 125, 126] that further contribute to stimulate fibroblast differentiation and proliferation, thus potentially linking inflammatory and fibrotic processes [127]. PDGF-*β* is produced by a wide array of lung cells, including macrophages, fibroblasts, epithelial and endothelial cells, [127, 128]. PDGF-*β* plays a primary role in the pathogenesis of IPF, figuring among profibrotic and proinflammatory molecules known to be critical in the pathogenesis of ILD, such as TGF-beta and TNF-alpha [127, 129]. It is interesting to note that the inhibition of PDGF tyrosine kinase receptor significantly attenuates the development of ILD in an experimental mouse model of pulmonary fibrosis [130]. Further studies regarding the role of PDGF in the development of RA-ILD are needed.

TGF-beta is produced by several cell types: macrophages, epithelial, endothelial and dendritic cells, and fibroblasts [127]. The profibrotic action of TGF-beta is mediated by the recruitment and activation of monocytes and fibroblasts, and by the induction of extracellular matrix deposition [127, 131]. TGF-beta also induces fibroblasts' differentiation into myofibroblasts, that represent the main source of extracellular matrix in lung fibrogenetic processes [132]. The role of chemokines (CXCL5, CXCL8, CXCL12, CXCL13) in the recruitment and organization of lung interstitial inflammatory infiltrate in RA-ILD is still largely unknown. These chemokines are secreted by macrophages, fibroblast and epithelial cells and act trough fibroblast recruitment and activation [127]. CXCL13 is a B-cells chemoattractant known to regulate lymphoid follicules organization within rheumatoid synovial tissue [116]. The exact role of this chemokine in the lung parenchima still needs to be clarified although it has been demonstrated that CXCL13 expression correlates with the extent of inducible bronchus-associated lymphoid tissue (iBALT) in patients with RA-ILD. This finding introduces a possible fascinating connection of pathogenetic mechanisms linking arthritis to extra-articular manifestations of RA. 

**Figure 1:**
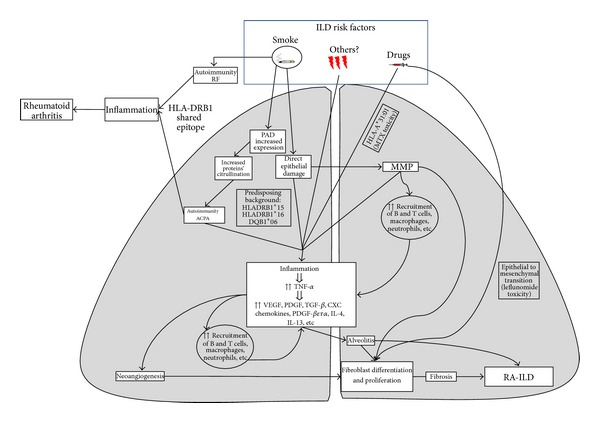
Possible pathogenetic mechanisms involved in the occurrence of interstitial lung disease in rheumatoid arthritis. ILD: interstitial lung disease, RF: rheumatoid factor, ACPA: anticyclic citrullinated peptide antibodies, VEGF: vascular endothelial growth factor, PDGF: platelet derived growth factor, PAD: peptidylarginine deiminase, and MTX: methotrexate, MMP: metalloproteinase.

Other mediators involved in this multi-step pathogenic cascade include matrix metalloproteinases, originated from damaged epithelia, that perpetuate this crosstalk between inflammatory and fibrotic processes, by the enhancement of cellular recruitment (B and T cells, macrophages, and neutrophils) and by the production of additional profibrotic mediators. Angiogenesis, induced by vascular endothelial cell growth factor and by the proinflammatory *milieu, *is also strictly connected with different phases of the pathological processes of inflammation and fibrosis (Figure 1).

## 6. RA-ILD Diagnosis 

Although some patients with proven RA-ILD can be asymptomatic, the majority present with exertional dyspnoea and dry cough. Pleuritic chest pain, fever, haemoptysis, and tachypnoea are also common. Bibasal crackles on chest examination are the most frequent finding [9, 31]. 

Plain chest radiography mainly reveals reticular and fine nodular opacities. These findings are usually more concentrated in the lower lung zones. Chest radiography has a low sensitivity for detection of ILD and can be normal in early stages [9]. In the majority of patients pulmonary function tests (PFT) demonstrate a restrictive defect with low forced vital capacity (FVC), low total lung capacity (TLC) with or without low diffusion capacity of the lung for carbon monoxide (DLCO), and hypoxemia at rest or on exertion [9, 31]. Decreased DLCO has been described in up to 40% of RA patients without signs or symptoms of lung disease [9] and is reported to be the most sensitive test for predicting the presence of ILD on HRCT [133]. 

Bronchoalveolar lavage (BAL) is not routinely used as a diagnostic modality in RA-ILD because BAL changes may be seen even in the absence of ILD. BAL characteristics in RA-ILD patients show a predominance of neutrophils and macrophages [31]. The analysis of BAL fluid may not be helpful to distinguish between different subtypes of RA-ILD; however, a slightly higher percentage of neutrophils (>4%) [134] may be more frequent in UIP. Lymphocytosis (>18%) is more common in NSIP and OP [31]. Nevertheless, the results of BAL cellularity may play a prognostic role, with neutrophilia associated with more advanced disease involvement and decreased response to therapy [134, 135]. BAL may also be useful in excluding infectious processes. 

HRCT has been accepted as the standard noninvasive method of diagnosing and following ILD in patients with RA [136]. The results of HRCT have been shown to correlate closely with those of open lung biopsy [13, 135]. The most frequent HRCT findings detected in different subtypes of RA-ILD are presented in Table 2. High prevalence of enlarged mediastinal lymphnodes in patients with ILD is described [136]. 

Surgical lung biopsy provides the best means of establishing a histopathological diagnosis. However, because of the potential risks associated with this procedure, many patients are diagnosed without pathological confirmation. Usually, video-assisted thoracoscopic surgery (VATS) is preferred to open-lung biopsy. It is important to note that transbronchial biopsies are not useful in the diagnosis, with the exception of DAD/AIP, and occasionally organizing pneumonia OP/COP [29]. Histological changes in RA-ILD are very similar to those seen in IPF [9] and are summarized in Table 2.

The individuation of biomarkers to be used in the clinical setting to guide diagnosis and response to treatment is still ongoing. Increasing attention has been paid to a serologic marker of pulmonary disease, KL-6 (Krebs von den Lungen-6, a high molecular weight glycoprotein expressed on proliferated type 2 alveolar pneumocytes and epithelial cells), that is known to occur in patients with interstitial pneumonia, hypersensitivity pneumonitis, tuberculosis, sarcoidosis, and pulmonary alveolar proteinosis. It has also been reported that KL-6 is elevated in RA-ILD [30, 137]. KL-6 can be used as a useful marker reflecting the severity of pulmonary fibrosis, the grade of alveolitis, and the extent of HRCT lesions and for detecting active and progressive lung disease in RA-ILD; however, KL-6 may not be very sensible in detecting early stages of lung disease [137]. Oyama et al. [138] demonstrated that KL-6 is elevated in 88.9% of patients with active ILD and only in 0.6% of RA patients without active interstitial disease. RA-ILD patients present high levels of proliferative potential colony-forming cells (HPP-CFCs) in peripheral blood compared to those without ILD [139]. High levels of anti-cytokeratin 19 [140], IL-1, anti-IL1 antibodies, and serum LDH are reported [141], but their clinical utility still has to be better defined.

## 7. Differential Diagnosis

Differential diagnosis can be very challenging considering that interstitial lung involvement is not exclusive of RA but can be a complication of several conditions in the setting of connective tissue diseases (CTD). Antisynthetase syndrome (ASS) is the prototypical example of these conditions. ASS is characterized by the occurrence of peripheral arthritis, myositis, interstitial lung disease (ILD), typical cutaneous manifestations (e.g., mechanic's hands), and Raynaud's phenomenon, together with the positivity of antisynthetase antibodies [20, 142]. In these patients, ILD is described in up to 95% [20]. Arthritis is frequently symmetrical, and joint erosions and ACPA positivity have been described also in ASS patients [143], making the differential diagnosis between RA and ASS particularly challenging. Some reports indicate that the onset of different manifestations may be spread out [144, 145] with patients presenting first with arthritis and then with ILD. In our experience on 18 ASS, arthritis was the first symptom in 5 cases; all patients developed ILD in a time interval ranging from 3 months to 13 years. 

Opportunistic infections, often connected with immunosuppressive treatments, particularly with biologic therapies, can mimick RA-ILD and can represent a real diagnostic challenge. Differential diagnosis in this setting can be even more troublesome in the case of patients with preexisting ILD [40]. Pathogens to consider as potential ILD mimickers are *Pneumocystis jiroveci, *fungal [40] and viral infections such as Epstein-Barr [146] and cytomegalovirus [147]. The main differential diagnosis based on radiological aspects is presented in Table 2. From the previously mentioned evidence it is clear that differential diagnosis is fundamental in distinguishing RA-ILD from different conditions that can be great mimickers such as ASS or infectious diseases. This process is fundamental in order to promptly start the appropriate treatment, with significant influence on patients' prognosis [20, 148].

## 8. RA-ILD Treatment

To date, little is known about optimal treatment for RA-ILD and whether the pattern of ILD may influence the response to immunosuppression [10]. For newly diagnosed patients, first-line treatment is generally based on high dose corticosteroids [149] that may be associated with immunosuppressants such as cyclophosphamide [14, 150] and azathioprine [14, 151]. Mycophenolate mofetil should be considered as a potentially useful treatment, considering its additional action on fibroblasts, endothelial cells, and smooth muscle cells [151, 152]. Similarly to ASS [20, 148], good results have been obtained with cyclosporine [153–156]. Despite the previously mentioned lung toxicity, cases of MTX and LEF efficacy in RA-ILD have been reported [157]. As stated in a previous section, the relationship between anti-TNF alpha agents and ILD is far from being elucidated, and the role of rituximab has been recently questioned after the first reports of drug-induced ILD. Data are scarce regarding other biological therapies such as abatacept or tocilizumab. This latter improved RA-ILD in one single case report [158], whereas other authors addressed ILD occurrence or exacerbation following tocilizumab therapy [159, 160].

## 9. Conclusions

Although ILD is a well-established EAM of RA with a substantial impact on prognosis, it is evident that several aspects are far from being fully elucidated and are still widely discussed. In particular, we should clarify the cascade of events underlying the occurrence of ILD, by linking all etiologic and pathogenetic steps, starting from established risk factors (such as smoking) and resulting in the ultimate pulmonary manifestation (lung fibrosis). The comprehension of these passages is mandatory in order to improve the therapeutic approach of RA-ILD and to minimize the risk of ILD development in potentially predisposed patients.

## Figures and Tables

**Table 1:** Histologic and clinical classification of IIPs, applicable to RA-ILD (adapted from [29–31]).

Histologic patterns	Clinical-radiological-pathologic diagnosis
Usual interstitial pneumonia (UIP)	(Idiopathic) pulmonary fibrosis/(cryptogenic) fibrosing alveolitis (IPF/CFA)
Nonspecific interstitial pneumonia (NSIP)	Nonspecific interstitial pneumonia (NSIP)
Organizing pneumonia (OP)	Organizing pneumonia (preferred definition) = Bronchiolitis obliterans organizing pneumonia (OP = BOOP)
Diffuse alveolar damage (DAD)	Acute interstitial pneumonia (AIP)
Respiratory bronchiolitis (RB)	Respiratory bronchiolitis interstitial lung disease (RB-ILD)
Desquamative interstitial pneumonia (DIP)	Desquamative interstitial pneumonia (DIP)
Lymphoid interstitial pneumonia (LIP)	Lymphoid interstitial pneumonia (LIP)

**Table 2:** Histological and radiological patterns of RA-ILD [9, 10, 29–31].

Pattern	Histology	CT features	CT differential diagnosis
UIP	Subpleural and peripheral fibrosis. Fibroblastic foci, lymphoid aggregates with germinal centres and honeycombing are characteristic. Mild inflammation; architectural destruction.	Peripheral, subpleural, basal reticulation, *and *honeycombing Traction bronchiectasis, architectural distorsion, GGO (less diffuse). Subpleural *lines *	IPF, other collagen vascular diseases, hypersensitivity pneumonitis (micronodules and sparing of lung bases), sarcoidosis, asbestosis (pleural thickening).

NSIP	Uniform interstitial involvement; various degrees of fibrosis and/or inflammation. Lymphoid aggregates. Rare honeycombing	Bilateral, symmetrical, patchy, mainly basal *GGO*, possible reticulation, traction bronchiectasis, irregular lines, or consolidation. Little or no honeycombing (in fibrosing NSIP).	UIP, DIP, COP, hypersensitivity pneumonitis, and HIV-associated interstitial lung disease.

OP	Connective tissue plugs within small airways and air spaces (Masson bodies). Little or no inflammation or fibrosis.	*Patchy and multiple airspace consolidation*, mainly basal, peripheral, or peribronchovascular. Air bronchograms can be seen. Possible associated GGO or centrilobular nodules.	Infections, vasculitis, sarcoidosis, alveolar carcinoma, lymphoma, eosinophilic pneumonia, NSIP, and COP.

DAD	(i) Acute phase: hyaline membranes, edema. (ii) Organizing phase: airspace and interstitial organization	(i) Acute phase: progressive, patchy, or diffuse *GGO *and dependent consolidation, often with lobular sparing (ii) Organizing phase: reticulation, traction bronchiectasis, and architectural distorsion.	Hydrostatic edema, pneumonia, eosinophilic pneumonia, and ARDS (but more symmetrical and lower lung zones)

DIP	Extensive macrophage accumulation in the distal air spaces. Mild interstitial involvement.	Patchy *GGO*, basal, and peripheral. Microcystic changes within GGO, reticular lines.	RB-ILD, hypersensitivity pneumonitis, sarcoidosis, and *Pneumocystis jiroveci *pneumonia.

RB-ILD	Bronchiolocentric macrophage accumulation. Mild bronchiolar fibrosis	Diffuse/upper lobes distribution, centrilobular nodules, bronchial wall thickening, and patchy GGO.	DIP, NSIP, and hypersensitivity pneumonitis

LIP	Bronchiolocentric lymphoid tissue hyperplasia	Diffuse, GGO, centrilobular nodules, septal and bronchovascular thickening, thin-walled *cysts,* and lymph node enlargement.	Sarcoidosis, lympangitic carcinoma, and Langherans' cell histiocytosis

GGO: ground glass opacities; UIP: usual interstitial pneumonia, NSIP: non-specific interstitial pneumonia, OP: organizing pneumonia; COP: cryptogenic organizing pneumonia; DAD: diffuse alveolar damage; DIP: desqumative interstitial pneumonia; RB-ILD: respiratory bronchiolitis-associated interstitial lung disease; LIP: lymphoid interstitial pneumonia; IPF: idiopathic pulmonary fibrosis.

## Glossary

ACPA: Anticyclic citrullinated peptide antibodies
AIP: Acute interstitial pneumonia
ASS: Antisynthetase syndrome
ATS/ERS: American Thoracic Society/European Respiratory Society
BAL: Bronchoalveolar lavage
BOOP: Bronchiolitis obliterans organizing pneumonia (recently classified as OP)
CFA: Cryptogenic fibrosing alveolitis
COP: Cryptogenic organizing pneumonia
CTDs: Connective tissue diseases
DAD: Diffuse alveolar damage
DIP: Desquamative interstitial pneumonia
DLCO: Diffusion capacity of the lung for carbon monoxide
DMARDs: Disease modifying antirheumatic drugs
EAMs: Extra-articular manifestations
EMT: Epithelial-mesenchymal transition
FVC: Forced vital capacity
GGO: Ground glass opacities
HRCT: High resolution computed tomography
IIPs: Idiopathic interstitial pneumonias
IL-13: Interleukin-13
IL-4: Interleukin-4
IL-6: Interleukin-6
ILD: Interstitial lung disease
IPF: Idiopathic pulmonary fibrosis
KL-6: Krebs von den Lungen-6
LEF: Leflunomide
LIP: Lymphoid interstitial pneumonia
MMP: Metalloproteinase
MTX: Methotrexate
NSIP: NonSpecific Interstitial Pneumonia
OP: Organizing pneumonia (preferred definition to BOOP)
PAD: Peptidylarginine deiminase
PDGF-*β*: Platelet derived growth factor-*β*
PFT: Pulmonary function tests
RA: Rheumatoid arthritis
RA-ILD: Interstitial lung disease in rheumatoid arthritis
RB-ILD: Respiratory bronchiolitis interstitial lung disease
RF: Rheumatoid factor
SE: Shared epitope
TGF-*β*: Transforming growth factor-*β*
TNF: Tumor necrosis factor
TLC: Total lung capacity
UIP: Usual Interstitial Pneumonia
VEGF: Vascular endothelial growth factor.
